# The HIV Matrix Protein p17 Promotes the Activation of Human Hepatic Stellate Cells through Interactions with CXCR2 and Syndecan-2

**DOI:** 10.1371/journal.pone.0094798

**Published:** 2014-04-15

**Authors:** Barbara Renga, Daniela Francisci, Elisabetta Schiaroli, Adriana Carino, Sabrina Cipriani, Claudio D'Amore, Angelo Sidoni, Rachele Del Sordo, Ivana Ferri, Monica Lucattelli, Benedetta Lunghi, Franco Baldelli, Stefano Fiorucci

**Affiliations:** 1 Department of Experimental and Clinical Medicine, University of Perugia, Perugia, Italy; 2 Department of Experimental Medicine and Biochemical Sciences, University of Perugia, Perugia, Italy; 3 Department of Experimental Medicine and Biochemical Sciences, Section of Anatomic Pathology and Histology, University of Perugia, Perugia, Italy; 4 Department of Life Sciences, University of Siena, Siena, Italy; Rutgers, The State University of New Jersey, United States of America

## Abstract

**Background:**

The human immunodeficiency virus type 1 (HIV-1) p17 is a matrix protein involved in virus life's cycle. CXCR2 and Syndecan-2, the two major coreceptors for the p17 protein, are expressed in hepatic stellate cells (HSCs), a key cell type involved in matrix deposition in liver fibrotic disorders.

**Aim:**

In this report we have investigated the *in vitro* impact of p17 on HSCs transdifferentiation and function and underlying signaling pathways involved in these processes.

**Methods:**

LX-2 cells, a human HSC line, and primary HSC were challenged with p17 and expressions of fibrogenic markers and of p17 receptors were assessed by qRT-PCR and Western blot. Downstream intracellular signaling pathways were evaluated with qRT-PCR and Western blot as well as after pre-treatment with specific pathway inhibitors.

**Results:**

Exposure of LX2 cells to p17 increases their contractile force, reshapes the cytoskeleton fibers and upregulates the expression of transdifferentiation markers including αSMA, COL1α1 and endothelin-1 through the activation of Jak/STAT and Rho signaling pathways. These effects are lost in HSCs pre-incubated with a serum from HIV positive person who underwent a vaccination with a p17 peptide. Confocal laser microscopy studies demonstrates that CXCR2 and syndecan-2 co-associate at the plasma membrane after exposure to p17. Immunostaining of HIV/HCV liver biopsies from co-infected patients reveals that the progression of liver fibrosis correlates with a reduced expression of CXCR2.

**Conclusions:**

The HIV matrix protein p17 is pro-fibrogenic through its interactions both with CXCR2 and syndecan-2 on activated HSCs.

## Introduction

In the era of effective antiretroviral therapy (ART), liver disease is the second most common cause of death among persons with human immunodeficiency virus (HIV) infection and a strong association between immunodeficiency and risk of liver-related death, exists [1], [2]. In addition, hepatitis B and C virus (HBV and HCV) and non-alcoholic steatohepatitis (NASH) are found frequently in HIV infected persons greatly increasing the burden for liver diseases [3], [4]. The mechanisms that drives liver injury in HIV infected persons are several and include both direct and indirect pathways. To date, amongst the HIV proteins, only the HIV envelope protein gp120 has been demonstrated to exert a direct profibrogenic action on humans Hepatic Stellate cells (HSCs), thus identifying a direct mechanism possibly linking HIV infection with liver fibrogenesis via envelope proteins [5]–[7]. Indeed, the HIV envelope protein gp120 directly acts on HSCs by binding and activating both CCR5 or CXCR4 receptors (the two major HIV co-receptors) and syndecans [5]–[8]. Syndecans are type I transmembrane cell surface heparan sulfate proteoglycans (HSPGs) that function as co-factors in cell-cell adhesion, in linking cells to ligands in the extracellular matrix, and in the binding of cellular growth factors [9], [10]. They also function as co-receptors for HIV-1 entry into primary target cells [11], [12]. Indeed, the enzymatic removal of cell surface heparan sulfates or the addition of soluble heparan sulfates, while leaving CD4 and chemokine receptors (CCR5 and CXCR4) unchanged, drastically reduces HIV-1 adhesion to and entry into specific target cells, including CD4+ T cells, Hela cells and macrophages [11].

The HIV matrix protein p17 is a structural protein that plays important functions in the viral replication cycle such as the recruitment of the viral surface/transmembrane gp120/gp41 envelope protein complex into virions [13] as well as the targeting of Pr55Gag proteins to their assembly sites at the plasma membrane of infected cells [14], [15]. The p17 exerts its biological activity on immune cells (T lymphocytes, monocytes and dendritic cells) upon interaction with the IL-8 receptors, CXCR1 and CXCR2 [16]. Furthermore, an interaction between p17 and syndecans (specifically syndecan-2 and -4) has been documented in HeLa cells and in human activated CD4+ T cells.^16,17^ The intracellular signal activated by p17 upon interaction with the IL8 receptors or syndecans involves the activation of both Rho/ROCK1 and JaK/STAT1 pathways [17]–[19].

Hepatic fibrosis and cirrhosis are chronic scarring processes of the liver which associate with increased and altered deposition of extracellular matrix (ECM). In the setting of chronic liver injury, HSCs undergo a process of trans-differentiation from a resting, fat-storing phenotype to a myofibroblast-like phenotype characterized by expression of fibroblastic cell markers such as α-smooth muscle actin (α-SMA). Activated HSCs release increased amounts of ECM components, such as α1-collagen type I, that contribute significantly to the fibrotic changes in cirrhosis [20], [21]. In addition, activated HSCs gain contractile phenotype and is well established that the contractile force generated by HSCs contributes to the regulation of sinusoidal blood flow and the development of portal hypertension [22]. A number of vasoactive molecules can trigger contractile response in HSCs with endothelin-1 being the most potent constrictor [23], [24].

The aims of the present study were to investigate the effect of p17 on HSC activation, collagen-I and endothelin-1 expression through interactions with both CXCR2 and syndecan-2 and to explore underlying intracellular pathways involved in these interactions. Present results demonstrate that p17 pirates the IL-8 receptors and syndecans to modulates HSCs function. Of clinical relevance we demonstrate that anti-p17 antibodies obtained from a patient included in an anti-p17 vaccination trial [25] protect HSCs from activation caused by the viral protein.

## Results

### LX2 and primary HSCs express CXCR2 and syndecan-2 and their stimulation with p17 promotes expression of fibrogenic markers

**Figure 1:**
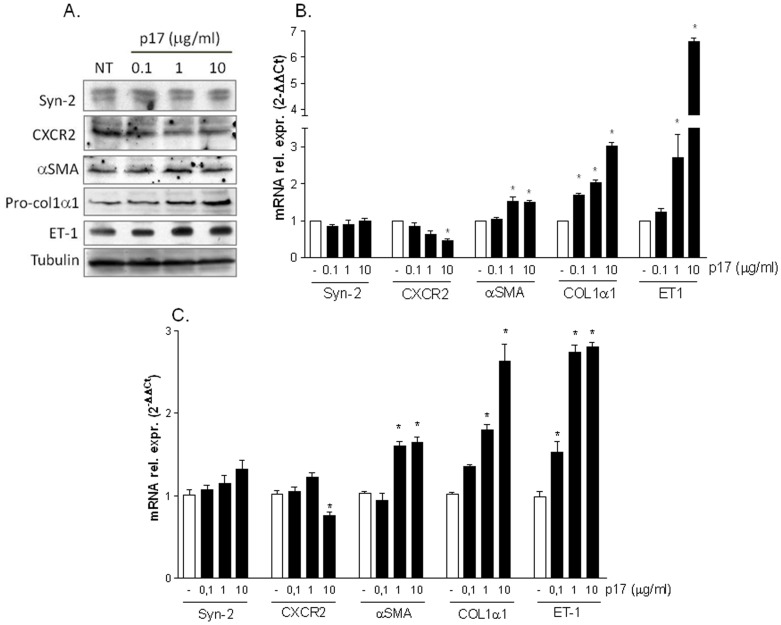
Effect of HIV p17 on stellate cell activation. (A–B) Serum starved LX2 cells were stimulated with 0.1, 1 and 10 µg/ml p17 for 18 hours. (A) Immunoblot of syndecan-2 (Syn-2), CXCR2, αSMA, pro-col1α1, endothelin-1 (ET-1) and Tubulin. (B) qRT-PCR of syn-2, CXCR2, αSMA, collagen-I and ET-1. Data represent the mean of 4 experiments. Values are normalized relative to HPRT mRNA and are expressed relative to those of untreated cells, which were arbitrarily set to 1. *P<0.05 versus not treated cells. (C) Serum starved human primary hepatic stellate cells were stimulated with 0.1, 1 and 10 µg/ml p17 for 18 hours. At the end of stimulations relative mRNA expression of syn-2, CXCR2, αSMA, collagen-I and ET-1 was evaluated by Real-Time PCR. Data represent the mean of 4 experiments. Values are normalized relative to HPRT mRNA and are expressed relative to those of untreated cells, which were arbitrarily set to 1. *P<0.05 versus not treated cells.

Since the HIV matrix protein p17 exerts its pro-inflammatory effects through the binding with CXCR2 and syndecan-2 [16], [17], [19], we have first examined whether LX2 cells, a human immortalized HSC line, express these two proteins. As shown in Figure 1 A and B, LX2 cells express CXCR2 and syndecan-2 as demonstrated by Western blotting analysis and RT-PCR. However, stimulation with escalating doses of p17 (from 0.1 to 10 µg/ml) resulted in a down-regulation of the expression of CXCR2 in these cells. Treating LX2 cells with p17 also resulted in a significant increase in the expression of collagen-I and endothelin-1 as confirmed by both Western blotting and RT-PCR while the induction of α-SMA was weak and did not changes significantly in cells exposed to 1 or 10 µg/ml of p17 (Fig.1 A, B and Fig. S1).

To extend the relevance of these observations to the pathophysiology of human stellate cells, primary stellate cells were stimulated with escalating doses of p17 (from 0.1 to 10 µg/ml) and the relative mRNA expression of CXCR2, syndecan-2, collagen-I, α-SMA and endothelin-1 examined by Real-Time PCR. Results from these experiments confirmed that primary human HSCs express CXCR2 and syndecan-2 and that the stimulation of these cells with p17 results in an induction of collagen-I, α-SMA and endothelin-1 comparable to tha observed in LX2 cells. Similarly to LX2 cells, exposure of human primary HSCs to 10 µg/ml p17 resulted in a significant downregulation of CXCR2 mRNA (Fig. 1C).

### Stimulation of LX2 cells with p17 reshapes cytoskeleton fibers

**Figure 2:**
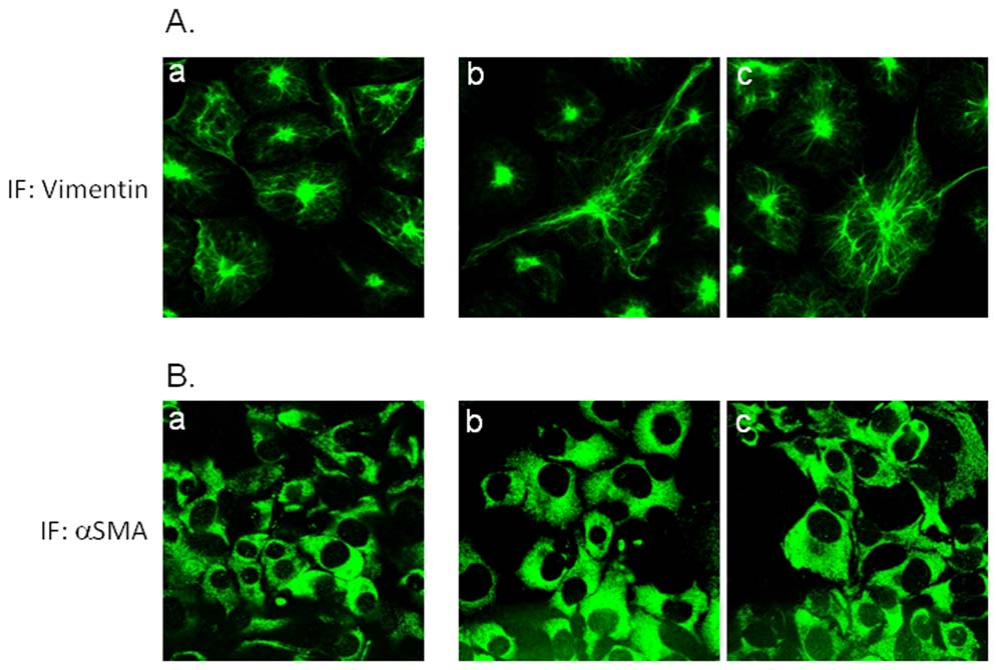
Effect of p17 on expression and distribution of cytoskeleton fibers in LX2 cells. Confocal immunofluorescence of vimentin (A) and α-SMA (B) in LX2 cells left not treated (a) or stimulated with 2 µg/ml p17 for 24 hours (b–c).

During the process of transdifferentiation HSCs undergo changes in the composition and amounts of cytoskeleton fibers. In particular, protein expression of focal adhesion protein (such as α-SMA and vinculin) as well as that of intermediate filaments (such as desmin and vimentin) changes during HSC activation [21], [26]–[27]. Thus, we have investigated the expression of vimentin and α-SMA in LX2 cells stimulated 24 h with p17 by confocal immunofluorescence. As shown in Figure 2, exposure of LX2 cells to p17 resulted in an increased organization of intermediate filaments as detected by confocal analysis of vimentin (Fig. 2A), while minor changes in α-SMA staining was seen in p17 treated cells in comparison with not stimulated cells (Fig. 2B).

### Pro-fibrogenic effects of p17 were neutralized by therapeutical vaccination

**Figure 3:**
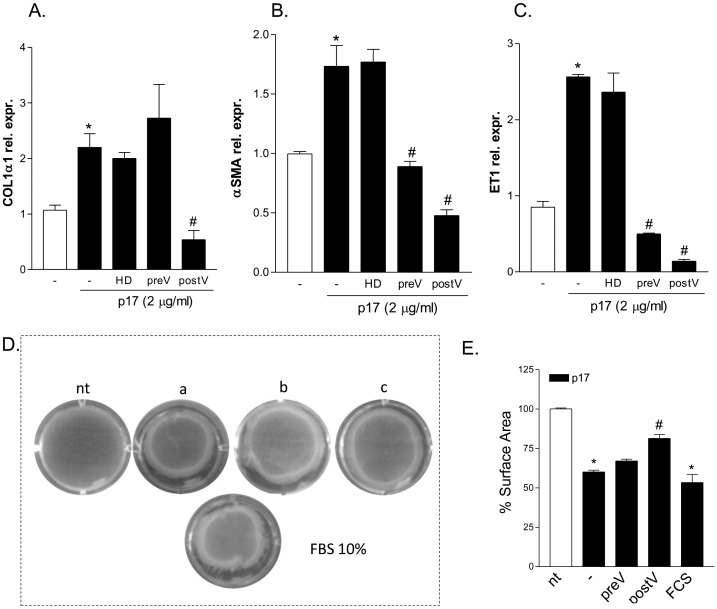
Blocking antibodies against p17 inhibit LX2 activation. (A–C) Serum starved LX2 cells were pre-incubated 2 h with the serum taken from and healthy donor (HD) or with the serum of an HIV patient taken before (preV) and after (postV) the vaccination with a recombinant p17 peptide, diluted 1∶100 in culture medium, followed by additional treatment with 2 µg/ml p17 for 18 hours. qRT-PCR of Col1α1 (A), αSMA (B) and ET-1. Values are normalized relative to HPRT mRNA and are expressed relative to those of untreated cells, which were arbitrarily set to 1. *P<0.05 versus not treated cells. #P<0.05 versus p17 stimulated cells. (D) Hydrated collagen lattices, photographed 18 hours after stimulation of LX2 with 10 µg/ml p17 alone (a) or in the presence of the serum of an HIV patient taken before (b) and after (c) the vaccination with a recombinant p17 peptide, diluted 1∶100 in culture medium. Contraction of LX2 cells in response to 10% FBS was also evaluated after 6 h incubation. (E) Mean percent gel surface area of collagen gels compared with initial surface area. Data are mean ± SE of 3 experiments. *P<0.05 versus control cells. #P<0.05 versus p17 stimulated cells. (nt: not treated cells).

Having shown that the HIV matrix protein p17 regulates the expression of proteins involved in HSC functions, such as collagen deposition and contraction, we have next investigated whether these effects could be reversed using the serum of an HIV positive patient who underwent a vaccination with a p17 peptide [18], [25]. As illustrated in Figure 3A–C, exposure of LX2 cells to p17 neutralizing antibodies retained in the sera of a vaccinated patient completely abrogates the effects of p17 in terms of induction of collagen-I, α-SMA and endothelin-1. Of interest, p17 mediated induction of α-SMA and endothelin-1, but not that of collagen-I was almost completely reversed even by using the serum taken before vaccination indicating that p17 effects could be neutralized with minimal amount of antibodies. As a control for this experiment we used a serum obtained from an HIV-negative subject. As shown in Figure 3A–C, the serum from this subject failed to block the fibrogenic effects exerted by p17 on LX2 cells (Fig. 3A–C).

Since endothelin-1 is a well characterized molecule involved in HSC contraction [23] and p17 treatment associates with up-regulation of endothelin-1 mRNA and protein, we then investigated whether the contractile response of LX2 cells to p17 was enedotelin-1 dependent. To measure the effect of p17 on the contraction of LX2 cells, we used the established assay of hydrated collagen gels [28]. As shown in Figure 2 D, exposure of gel for 6 hour to 10% FBS (positive control), resulted in a significant decrease of the collagen surface area. Compared with positive control, the treatment with p17 caused a similar reduction of the collagen surface area after 18-hour incubation. Interestingly, pre-treatment of LX2 cells with serum taken before vaccination failed to reverse the p17 mediated LX2 contraction while the post-vaccination serum almost completely abrogated these effects (Fig. 2E). All together these data indicated that anti-p17 immune-neutralization leads to a specific reversion of p17 mediated activities on LX2 cells.

### p17 induction of collagen-I and α-SMA is CXCR2 dependent and occurs via Rho/ROCK-1 pathway

**Figure 4:**
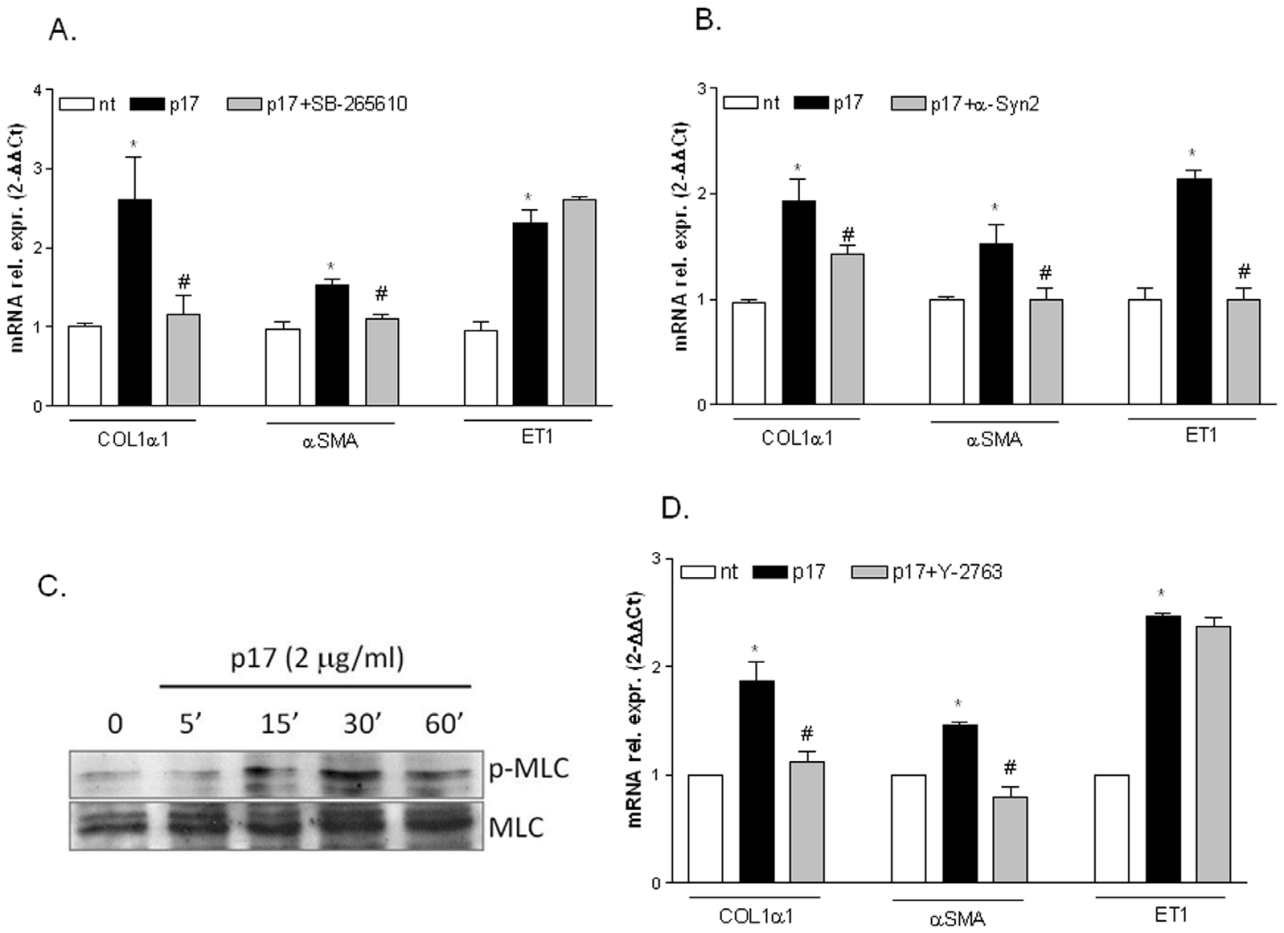
p17 activates CXCR2/Rho pathway on LX2 cells. (A) Serum starved LX2 cells were pre-incubated for 2 h with 100 nM SB265610 followed by additional treatment with 2 µg/ml p17 for 18 hours. qRT-PCR of collagen-I, αSMA and ET-1. Values are normalized relative to HPRT mRNA and are expressed relative to those of untreated cells, which were arbitrarily set to 1. *P<0.05 versus not treated cells. #P<0.05 versus p17 stimulated cells. (B) Serum starved LX2 cells were pre-incubated for 2 h with 4 µg anti-syndecan-2 antibody followed by additional treatment with 2 µg/ml p17 for 18 hours. qRT-PCR of collagen-I, αSMA and ET-1. Values are normalized relative to HPRT mRNA and are expressed relative to those of untreated cells, which were arbitrarily set to 1. *P<0.05 versus not treated cells. #P<0.05 versus p17 stimulated cells. (C) Time course of phosphoMLC and total MLC expression in LX-2 cells treated with 2 µg/ml of p17. (C) LX2 cells were pre-incubated for 2 h with indicated concentrations of Rho kinase inhibitor Y-27632 prior to stimulation with 2 µg/ml p17 for 18 hour. qRT-PCR of collagen-I, αSMA and ET-1. Values are normalized relative to HPRT mRNA and are expressed relative to those of untreated cells, which were arbitrarily set to 1. *P<0.05 versus not treated cells. #P<0.05 versus p17 stimulated cells.

Since p17 signals through the binding with both the IL8 receptors and Syndecan-2, a membrane peptidoglycan, we have next investigated whether the effects of p17 on LX2 cells were CXCR2 and/or Syndecan-2 dependent. First, LX2 cells were pre-treated with a CXCR2 inhibitor (SB-265610) before exposure to p17. Results obtained by Real-Time PCR analysis demonstrated that the CXCR2 inhibitor effectively reversed the p17-mediated induction of collagen-I and α-SMA, while it failed in reversing the induction of endothelin-1 (Figure 4 A). In a second set of experiments, LX2 cells were pre-treated with a syndecan-2 antibody before exposure to p17. Results from these experiments demonstrated that the blockade of syndecan-2 reversed the p17-mediated induction of collagen-I, α-SMA and endothelin-1 (Figure 4 B). Since the intracellular signaling activated by CXCR2 involves the activation of Rho/ROCK-1 [17] and one of the major consequences of activation of Rho kinase is the phosphorylation of myosin light chains (MLC) [29], we have examined whether p17 induces the phosphorylation of MLC, which is critically involved in the contraction of HSC [30]. Results from these investigations demonstrated that exposure of LX2 cells to p17 increases the amount of phosphorylated MLC. In particular, the p17 mediated phosphorylation of MLC occurs after 15′ stimulation of LX2 cells and still remains constant up to 60 minutes (Fig. 4 C and Fig. S2–A). To further confirm that the pathway triggered by p17 involves the activation of Rho kinase we have pre-treated LX2 cells with a specific Rho kinase inhibitor (Y-27632) before exposure to p17. As illustrated in Figure 4 D, confirming the results obtained with the CXCR2 inhibitor SB-265610, the treatment of LX2 cells with the Rho inhibitor Y-27632 completely reversed the p17-mediated induction of collagen-I and α-SMA but not that of endothelin-1. All together these data demonstrated that p17 induction of collagen-I and α-SMA occurs via CXCR2 activation of Rho kinase and suggested that the p17-mediated up-regulation of endothelin-1 could take place with another additional mechanism which involves syndecan-2 activation.

### p17 stimulation of LX2 cells activates the Jak/STAT intracellular signaling

**Figure 5:**
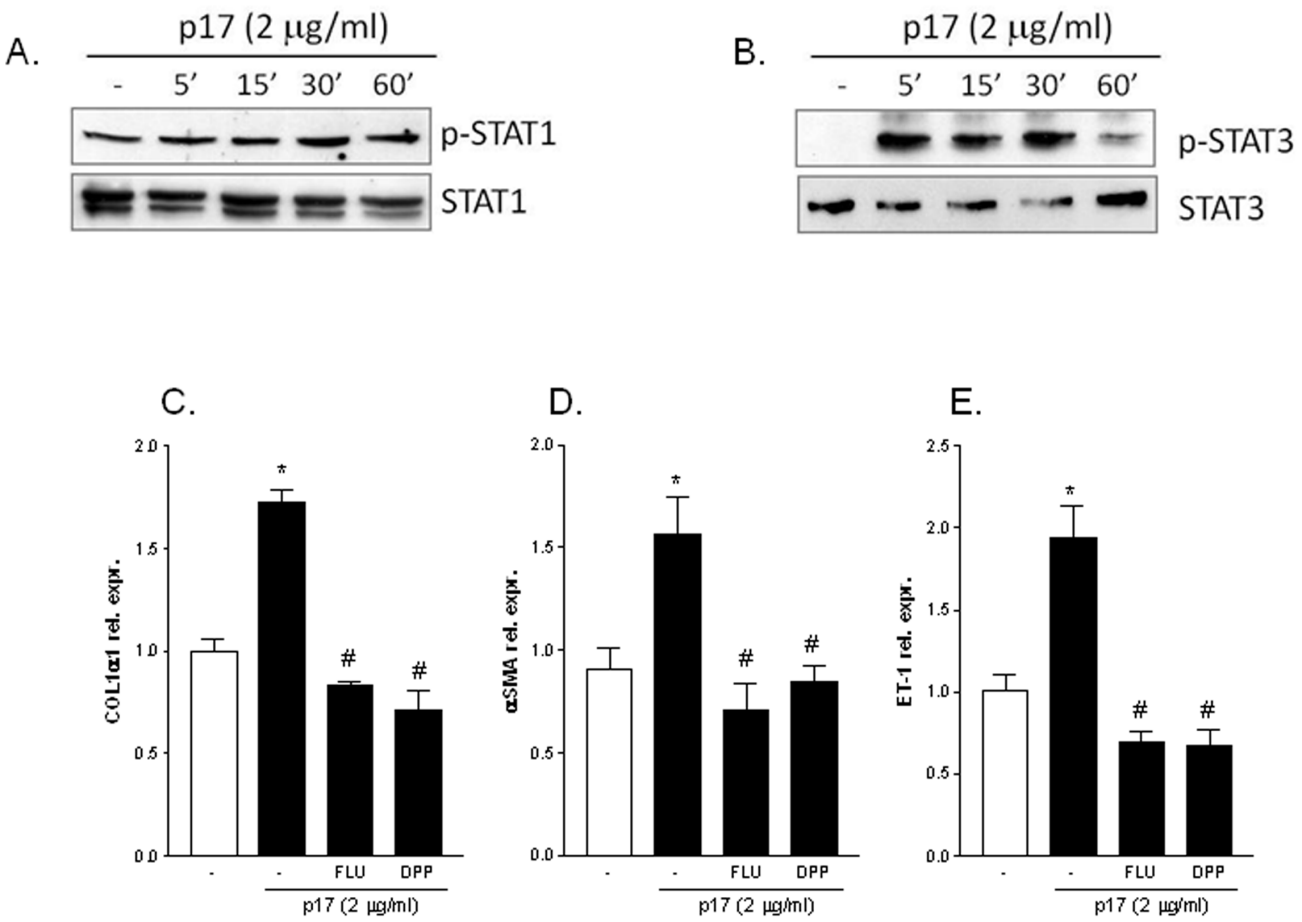
HIV matrix protein p17 activates JAK/STAT pathway on LX2 cells. (A) Time course of phosphoSTAT1 and total STAT1 expression in LX-2 cells treated with 2 µg/ml of p17. (B) Time course of phosphoSTAT3 and total STAT3 expression in LX-2 cells treated with 2 µg/ml of p17. (C–E) LX2 cells were pre-incubated for 2 h with the STAT1 inhibitor Fludarabine (5 µM) or with the STAT3 inhibitor 5,15 DPP (5 µM) prior to stimulation with 2 µg/ml p17 for 18 hour. qRT-PCR of collagen-I (C), αSMA (D) and ET-1 (E). Values are normalized relative to HPRT mRNA and are expressed relative to those of untreated cells, which were arbitrarily set to 1. *P<0.05 versus not treated cells. #P<0.05 versus p17 stimulated cells.

Since it has been well recognized that signal transducers and activators of transcription (STAT) proteins regulate the expression of HSC markers (i.e. endothelin-1 and collagen-I) [30]–[33] and the HIV matrix protein p17 signals even by activating the JaK/STAT pathway [18] we next investigated whether p17 induces phosphorylation of STAT proteins (i.e. STAT1 and STAT3) in LX2 cells. As illustrated in Figure 5 A and B, we found that exposure to p17 increases the level of phosphorylated STAT1 protein after 30 minutes as well as increases the level of phosphorylated STAT3 protein after 5 minutes of stimulation (Fig. 5A, B and Fig. S2 B and C). Thus, we next evaluated the involvement of STAT1 and STAT3 in the synergistic actions of p17 on LX2 by co-administering cells with both the STAT1 inhibitor fludarabine or the STAT3 inhibitor 5,15 DPP in the presence of p17. Results from RT-PCR demonstrated that both fludarabine and 5,15 DPP significantly reduced p17 mediated stimulation of collagen-I, α-SMA and endothelin-1 (Fig. 5 C–E).

### CXCR2 and syndecan-2 co-localize after stimulation of LX2 cells with p17

**Figure 6:**
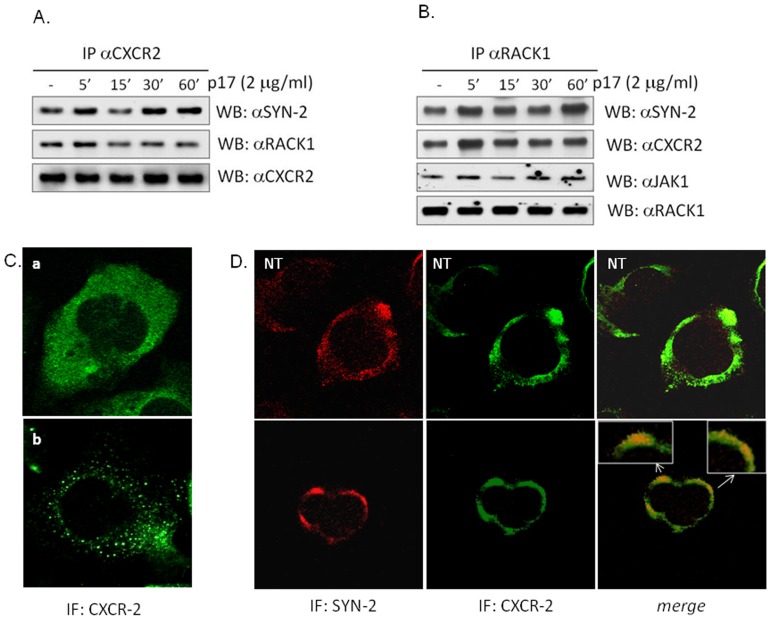
p17 drives the formation of a multiprotein complex containing CXCR-2, syn-2, RACK-1 and JAK-1 on LX2 cells. LX2 cells were serum starved and stimulated for 5, 15, 30 and 60 minutes with 2 µg/ml of p17. (A) Co-immuneprecipitation of CXCR2 with syndecan-2 and RACK-1. (B) Co-immuneprecipitation of RACK1 with syndecan-2, CXCR2 and JAK1. (C) Confocal immunofluorescence of LX2 cells left untreated (a) or exposed to p17 for 5 minutes and stained with anti-CXCR2 antibody. (D) Immunofluorescence analysis of syndecan-2 and CXCR2 localization on LX2 cells left untreated (NT) or stimulated 5 minutes with p17. Cells were stained with the indicated antibodies. Red: syn-2. Green: CXCR2. The red and green signals were electronically merged for co-localization analysis.

We next investigated the hypothesis that CXCR2 and syndecan-2 could interact as a result of their binding with p17. Results from immunoprecipitation experiments demonstrated the existence of a multiprotein complex between the proteins CXCR2, syndecan-2 and RACK1 which is clearly assembled already in basal conditions. Of interest, the interaction between these proteins increased after 5 min stimulation of LX2 cells with p17 (Fig. 6 A and Fig. S3 A and B). These results were further confirmed by immunoprecipitation experiments with RACK-1 antibodies. Indeed, results from these experiments not only confirmed that both CXCR2 and syndecan-2 interact with RACK1 after exposure of LX2 cells to p17 but also highlighted that RACK-1 interacts with JAK-1 (Fig. 6 B and Fig. S3 C–E).

The mutual relations between CXCR2 and syndecan-2 were further confirmed by analyzing LX2 cells at the confocal microscope. As illustrated in Figure 6 C, a different subcellular localization of CXCR2 between control and p17-treated cells has been found as resting LX2 cells exhibited both cell surface and cytoplasmic diffuse staining while p17-treated cells shown a “punctated vesicular-like” staining. Furthermore, results obtained making a double staining CXCR2/syndecan-2 demonstrated that these two proteins coexist in areas where the signals (red for syndecan-2 and green for CXCR2) are superimposed (Figure 6 D). All together these data demonstrated that the HIV matrix protein p17 promotes the formation of a multiprotein complex characterized by the proteins CXCR2, syndecan-2, RACK-1 and JAK-1.

### Progression of liver fibrosis correlates with a reduction of CXCR2 protein expression *in vivo*

**Figure 7:**
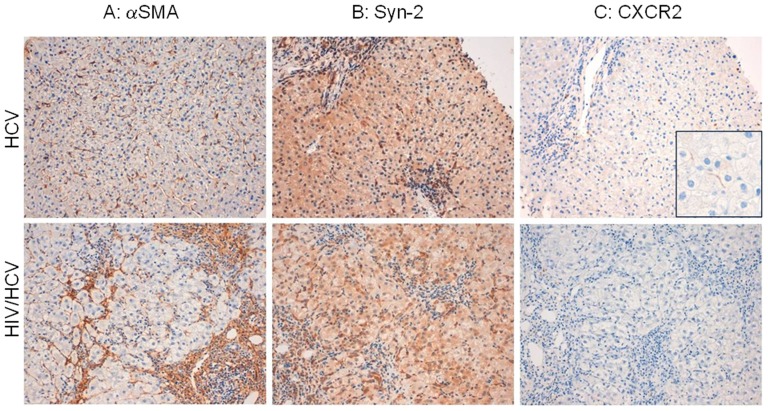
Progression of liver fibrosis is associated with a down-regulation of CXCR2 receptor. Liver biopsies from HIV/HCV coinfected and HCV monoinfected patients were serially sectioned and stained with the following antibodies: αSMA, syndecan-2 and CXCR2. (A) Patient n. 3 HCV positive. Diffusely and intensely sinusoidal walls staining with αSMA (Magnification 200x). Patient n.6 HIV/HCV positive. Focally and weakly sinusoidal walls staining with αSMA (Magnification 200x). (B) Patient n. 3 HCV positive and patient n. 6 HIV/HCV positive. In both cases strongly cytoplasmatic staining of stellate cells with syndecan-2 (Magnification 200x) (C) Patient n. 3 HCV positive. Focally and weakly sinusoidal walls staining with CXCR2 (Magnification 200x; inset 400x). Patient n.5 HIV/HCV positive. Absence of staining with CXCR2 (Magnification 200x)

To investigate if the above described in vitro observation has a clinical readout we have examined the expression of syndecan-2 and CXCR2 in liver biopsies obtained from HCV monoinfected and HCV/HIV co-infected patients with various degree of liver fibrosis (see Table 1 for staging of fibrosis). As shown in Figure 7 A and B, the investigation of the p17 co-receptors reveals a trend indicating that while protein expression of syndecan-2 did not change significantly during the progression of liver fibrosis (from mild to severe irrespective of viral status), that of CXCR2, which occurs primarily in neutrophils and along the hepatic sinusoids (inset of Figure 7 C), was greatly reduced in liver biopsies obtained from patients with severe fibrosis (Figure 7 C), thus confirming the *in vitro* observations that during the activation of HSCs the expression of CXCR2 is down-regulated.

**Table 1:** Fibrosis: Comparison of HCV and HIV/HCV Patients.

Patients	Age (years)	Staging of Fibrosis (Ishak score)	Sex M,F	HCV genotype
**HCV monoinfected**				
1	57	0	F	1
2	50	0	M	4
**3**	**77**	**0**	**M**	**1**
4	43	1	M	1
5	59	1	F	2
**HIV/HCV coinfected**				
**6**	**57**	**5**	**M**	**1**
7	52	3	F	1
8	40	0	F	1

*Immunostaining of liver biopsies from these patients are illustrated in Figure 6.

## Discussion

In the present study we have provided evidence that the HIV matrix protein p17 exerts profibrogenic effects on HSCs. This conclusion emerges from the following results: (i) LX2 cells and human primary HSCs express CXCR2 and syndecan-2, the two main plasma membrane receptors for p17; (ii) exposure of LX2 and HSCs to p17 drives the production of ECM components, such as α1-collagen type 1; (iii) p17 induces the reorganization of intermediate filaments such as vimentin and increases the contractile force of LX2 by inducing endothelin-1; (iv) p17 immune-neutralization completely abrogates the effects of p17 on HSCs contraction and induction of collagen-I, α-SMA and endothelin-1.

Results from the present study are consistent with hypothesis that p17 hijacks two alternative signaling pathways in LX2 cells i.e. the CXCR2/Rho pathway and the Jak/STAT pathway. Rho is a small, monomeric guanosine triphosphate-binding protein from the Ras superfamily [34]. The Rho kinase regulates downstream mediators, such as myosin light chain (MLC), through phosphorylation. Phosphorylated MLC (pMLC2) binds to actin and stabilizes the stress fibers necessary for migration and contraction [35]. Several studies have demonstrated that the Rho pathway is activated by several regulatory cytokines during fibrogenesis (i.e. TGFβ1 and PDGF) and is responsible for assembly of actin stress fibers, contractility and chemotaxis of HSCs [21]. Furthermore, inhibition of Rho kinase reduces MLC phosphorylation leading to a decreased activity of the promoter for myofibroblast marker α-SMA and to a reduced deposition of type I collagen [36]–[38]. Results presented in this study demonstrated that the treatment of LX2 cells with p17 results in a significant induction of MLC phosphorylation and that Y-27632, a selective inhibitor of Rho kinase, completely abrogates the profibrogenic effects exerted by p17 on these cells. Similarly, we found that while the pathway activated by CXCR2 was responsible for the p17 mediated induction of type I collagen and α-SMA, the inhibition of receptor and post-receptor checkpoints in the CXCR2 pathway, failed to modulate endothelin-1 gene expression. All together these results indicated that the CXCR2/Rho pathway regulates the expression of type I collagen and α-SMA and regulates contractile force by inducing the phosphorylation of MLC.

The contractile force of LX2 was only in part due to Rho activation of MLC. Indeed, our results support the notion that p17 might activate the JaK/STAT pathway, which in turn induces the transcription/expression of endothelin-1. Consistent with this view we have detected a marked activation of STAT1 and STAT3 after exposure of LX2 cells to p17. Of note, when LX2 cells were pre-incubated with STAT-1 and STAT3 inhibitors the effects exerted by p17 on endothelin-1, collagen-I and α-SMA were reduced. These results indicate that regulation of these effector functions by p17 take place through the activation of various, but partially convergent, pathways. Moreover, we found that the pre-incubation of LX2 cells with an anti-syndecan-2 antibody exerts the same regulatory effect of the STAT inhibitors. These results confirm previous observations that syndecans regulates the JaK/STAT signal transduction [39].

An important observation we have made in this study was that the exposure of both LX2 cells and primary human HSCs to p17 drives a robust reduction of CXCR2 expression while the expression of syndecan-2 remains unchanged. The negative regulation of CXCR2 expression that occurs in activated HSCs during their trans-differentiation is probably p17 independent. Indeed, it has been previously demonstrated that CXCR2 expression declines in a non-specific manner during LX2 trans-differentiation [40]. In particular, when LX2 cells were cultured in presence of 1% FBS (i.e. quiescent condition) they express much higher levels of CXCR2 compared to cells cultures with 10% FBS (i.e. activated condition) [40]. To provide translational information to this observation we have examined how the expression of p17 putative receptors changes with progression of liver fibrosis in HIV/HCV coinfected and HCV monoinfected patients. These immunohistochemical studies revealed that CXCR2 expression was non detectable in liver biopsies from patients with severe fibrosis while the expression of syndecan-2 and α-SMA remained stable, thus confirming our *in vitro* results that activation of HSCs, *per se*, causes the downregulation of the CXCR2 receptor.

Confirming a previous study [18], we have shown that exposure of LX2 to the HIV matrix protein p17 drives the formation of a multiprotein complex between the IL-8 receptor CXCR2, syndecan-2, RACK1 and JAK1. These results came from immunoprecipitation experiments and were further confirmed by confocal studies which provide evidence that CXCR2 colocalizes with syndecan-2 after 5 minutes exposure of LX2 cells to p17.

An important finding of the present study is the demonstration that the serum of an HIV positive patient who underwent a vaccination program [25] with a p17 protein contains anti-p17 antibodies which effectively neutralize the biological effects of the viral protein. The fact that a serum taken from the same HIV infected person before vaccination, but not a serum from an HIV negative healthy subject, was partially effective in reversing some effects of p17, is consistent with the presence of low title p17 neutralizing antibodies [18]. Since liver fibrosis is an important complication of long-term infection in HIV infected persons, present results suggest a possible clinical application of anti-p17 vaccination in the prevention of liver complication during HIV infection.

In conclusion, we have demonstrated that the HIV matrix protein p17 hijacks the CXCR2 and syndecan-2 signaling pathways and exerts profibrogenic effects in HSCs in vitro. Our results therefore, provide an additional explanation for liver fibrosis in HIV infected persons. Indeed, the presence of circulating p17 particles in HIV/HCV coinfected or in HIV monoinfected patients may not only be responsible for local activation and recruitment of inflammatory cells, but also for the direct activation of HSCs in the liver.

## Materials and Methods

### Chemicals

Y-27632, fludarabine, 5,15 DPP and SB-265610 were obtained from Sigma (St. Louis, MO, USA). Recombinant HIV-p17 protein was provided by Medestea (Torino, Italy).

### Cell culture and stimulation

LX-2 cells, an immortalized human hepatic stellate cell line, were cultured at 37°C in an atmosphere of 5% CO2 in Dulbecco's modified minimal essential medium (Gibco BRL Life Technologies, Rockville, MD) containing 2% fetal bovine serum (FBS), 1% L-glutamine and 1% penicillin/streptomycin.

For studies focused on determining the impact of HIV p17 protein on the expression of collagen-I, α-SMA, CXCR2, syndecan-2 and endothelin-1, cells were serum starved for 48 hours prior to challenge with 0.1, 1 and 10 µg/ml p17 for 18 hours. At the end of the incubation cells were lysed for isolation of total RNA and proteins.

To investigate the effect of the serum of an HIV patient who is subjected to vaccination with a recombinant p17 peptide, serum starved LX2 cells were pre-incubated 2 h with the serum of the patient taken before and after the vaccination, diluted 1∶100 in culture medium, followed by additional treatment with 2 µg/ml p17 for 18 hours. As a control, LX2 cells were also stimulated with a serum taken from an healthy donor. After stimulation cells were lysed for isolation of total RNA and proteins.

To investigate the effects of CXCR2 inhibitor SB-265610 (100 nM), Rho inhibitor Y-27632 (10 µM), STAT1 inhibitor fludarabine (5 µM), STAT3 inhibitor 5,15 DPP (5 µM) and syndecan-2 antibody (4 µg/well - Santa Cruz – sc-365624), 5×10^4^ LX2 cells were plated on a 6-well plates. After 48 hours of starvation cells were pre-incubated with the indicated concentrations of inhibitors for 2 hours followed by additional treatment with 2 µg/ml p17 for 18 hours. After stimulation, cells were lysed for isolation of total RNA and proteins.

### Primary human hepatic stellate cells

Human primary hepatic stellate cells were purchased from Innoprot (Barcellona, Spain). Cells were cultured were cultured at 37°C in an atmosphere of 5% CO2 in Stellate cell medium (Innoprot) added with 2% 2% fetal bovine serum (FBS), Stellate cell growth supplement (SteCGS – Innoprot) and 1% penicillin/streptomycin solution.

For studies focused on the impact of HIV p17 protein on the expression of collagen-I, α-SMA, CXCR2, syndecan-2 and endothelin-1, cells were serum starved for 24 hours prior to challenge with 0.1, 1 and 10 µg/ml p17 for 18 hours. At the end of the incubation cells were lysed for isolation of total RNA.

### Western Blot analysis

To investigate the effect of p17 on phosphorylation status of MLC, STAT1 and STAT3 total lysates were prepared by solubilization of cells in E1A lysis buffer (250 mM NaCl, 50 mM Hepes pH 7.0, 0.1% NP40, 50 mM EDTA) containing phosphatase and protease inhibitors. To investigate the effect of p17 on total protein expression of collagen-I, α-SMA, CXCR2, syndecan-2, endothelin-1 and tubulin, proteins were prepared for electrophoresis by boiling in 1X SDS Sample buffer (50 mM Tris HCl pH 6.8, 2.5% beta mercaptoethanol, 2% SDS, 10% glycerol). Following transfer to nitrocellulose membranes (Bio-Rad) proteins were detected with the following primary antibodies: procollagen type I (Santa Cruz - sc-8787), α-SMA (Santa Cruz – sc-53015), CXCR2 (Santa Cruz – sc-7304), syndecan-2 (Santa Cruz – sc-365624), endothelin-1 (Santa Cruz – sc-21625), phospho-MLC (thr18/ser19) (Santa Cruz – sc-12896), total MLC (Santa Cruz – sc-28329), α-tubulin (Sigma – T 6074), phosphoSTAT1(tyr701) (Cell Signaling - #9171), total STAT1 (Cell Signaling - #9172), phospho STAT3(tyr705) (Santa Cruz – sc-7993) and total STAT3 (Santa Cruz – sc-8019). Primary antibodies were detected with the horseradish peroxidase (HRP)-labeled secondary antibodies. Proteins were visualized by SuperSignal West Dura Extended Duration Substrate (Thermo Fisher Scientific Inc.) according to the manufacturer's instructions.

### Vaccination protocol

The HIV matrix protein p17 was immune-neutralized by incubation with sera from an HIV infected patient enrolled in a Phase 1 study designed to investigate the safety and immunogenicity of recombinant p17 peptide in HIV. The therapeutic vaccination was performed using a 20 amino acids peptide, named AT20-KLH (SGGELDRWEKIRLRPGGKKK). The vaccination protocol n. MED-AT20-001 Eudract Number 2008-001465-29 had been approved by the Ethical committee of Regione Umbria (Italy) on June 25, 2010 authorization n. 1558/10. Authorization for collecting and using blood samples from HIV infected persons for *ex vivo* testing was also granted by the ethical committee of Regione Umbria (Italy) on July 22, 2010 (authorization number CEAS 1654/20). An informed written consent was obtained from each participant to the study. The presence of anti-p17 antibodies in the blood of these subjects had been verified by micro-Elisa as previously described [18].

### Quantitative Real-Time PCR

Total RNA was extracted with 1 ml Trizol Reagent (Invitrogen) according to the manufacturer's instructions. One µg total RNA was reverse-transcribed using the enzyme Super Script II (Invitrogen) and quantitative Real time Polymerase Chain Reaction (qRT-PCR) was performed using primers indicated in Table 2. The PCR mixture was prepared by adding the following reagents: 50 ng cDNA, 0.2 µM of each primer and 12.5 µl of 2X SYBR Green qPCR master mix (Invitrogen) in a final volume of 25 µl. All reactions were performed in triplicate and the thermal cycling conditions were: 2 min at 95°C, followed by 40 cycles of 95°C for 20 s, 55°C for 20 s and 72°C for 30 s in iCycler iQ instrument (Biorad). The results of Real-Time PCR were normalized with the housekeeping gene HPRT and expressed as 2^−(ΔΔCt)^.

**Table 2:** Primers used for Real-Time PCR.

Gene	Forward	Reverse	Accession Number
**HPRT**	aagggtgtttattcctcatgga	ttgatgtaatccagcaggtcag	NM_000194.2
**type I collagen-αI**	acgtcctggtgaagttggtc	cagggaagcctctctctcct	NM_000088.3
**α-SMA**	acccacaatgtccccatcta	gaaggaatagccacgctcag	NM_001613.2
**endothelin-1**	agggctgaagacattatggaga	cctggtttgtcttaggtgttcc	NM_001955.4
**CXCR2**	gctctgactaccacccaacc	gctgggcttttcacctgtag	NM_001168298.1
**Syn-2**	gatgacgatgactacgcttctg	aggtgactttgtctgagcaggt	NM_002998.3

### Gel contraction assay

Contractility of LX2 was evaluated using collagen gel lattices on 12-well culture plates. Briefly, wells were filled with 3 ml PBS containing 1% bovine serum albumin for 1 hour at 37°C, washed twice with PBS, and air-dried. Type 1 bovine collagen (3 mg/mL; Sigma) was adjusted according to the manufacturer's instructions. Wells were filled with 1 ml collagen solution and incubated for 1 hour at 37°C to allow gelation. LX2 cells were trypsinized, suspended in DMEM (1×10^5^ cells/mL) supplemented with 1% fetal bovine serum and antibiotics, and plated on the collagen gels (2 mL cell suspension/well). After incubation overnight to allow cell attachment, LX2 were serum starved over-night. Gels were then detached from the plates. LX2 were pre-incubated for 2 hours with the serum of an HIV patient taken before and after vaccination with a p17 peptide (diluted 1∶100 in culture medium). After pre-incubation LX2 cells were stimulated for 18 hours with p17 (2 µg/ml). Culture medium containing 10% FBS was used as a positive control. The gels were photographed after 18 hours. Surface area of the collagen gels was measured using digital image analysis software.

### Immunoprecipitation protocol

To study the p17 intracellular signaling LX2 cells were serum starved over-night and then stimulated with p17 (2 µg/ml) for 5, 15, 30 and 60 minutes. After the stimulation, cells were washed 3 times with ice-cold PBS and lysed with an insulin syringe in 500 µl E1A lysis buffer (250 mM NaCl, 50 mM Hepes pH 7.0, 0.1% NP40, 5 mM EDTA). Lysates were incubated 20 minutes in ice, clarified by centrifugation at 12000 rpm for 20 minutes at 4°C and quantified with Bradford reagent (PIERCE). 200 µg total proteins were pre-cleared on a rotating wheel for 1 h at 4°C using protein A Sepharose beads (Amersham Biosciences). Immunoprecipitation was performed overnight at 4°C with the followings antibodies: 1 µg anti-RACK-1 antibody (Santa Cruz – sc-17754), 1 µg anti-CXCR2 antibody (Santa Cruz – sc-7304) or 1 µg anti-IgG as a negative control antibody in the presence of 40 µl of protein A Sepharose (Amersham Biosciences). The resultant immunoprecipitates were washed five times with 1 ml of lysis buffer and resuspended in 40 µl of 2X SDS Sample buffer (100 mM Tris HCl pH 6.8, 5% β-mercaptoethanol, 4% SDS, 20% glycerol). Anti CXCR2 immunoprecipitates were used for western blotting using the antibodies syndecan-2, RACK-1 and CXCR2. Anti RACK-1 immunoprecipitates were used for western blotting using the antibodies syndecan-2, RACK-1, JAK-1 (Santa Cruz – sc-7228) and CXCR2.

### Confocal Immunofluorescence

LX2 cells were plated in 2 chamber polystyrene vessels (1×10^5^ cell per chamber) previously coated with poly-l-lysyne (Sigma). Cells were serum starved overnight and then treated with p17 (2 µg/ml) for 5 minutes or 24 hours. After stimulation cells were washed in PBS and fixed with PFA 4% in PBS for 15 minutes. For CXCR2 and Syndecan double staining cells were incubated in a blocking solution containing BSA 6% in PBS. For vimentin and α-SMA single staining cells were incubated in PBS containing BSA 6% and triton 0,3%. Primary antibodies were incubated for 1 hour in PBS with the following dilutions: rat anti-human syndecan-2 (R&D systems) 1∶40; mouse anti CXCR2 (Santa Cruz) 1∶100; mouse anti vimentin (Dako) 1∶50; mouse anti α-SMA (Novus Biologicals) 1: 100. Cells were washed in PBS and then incubated with secondary antibodies Alexa Fluor 488 goat anti mouse IgG and Alexa Fluor 546 goat anti rat IgG (Life technologies) (1∶100 in PBS) for 30 minutes. After washing in PBS, slow fade gold antifade reagent was applied to slides and covered with cover slip.

### Immunohistochemistry of human liver biopsies

Human liver tissues used in the present study were obtained from needle biopsy previously performed in HCV-HIV coinfected patients and HCV-positive-HIV-negative patients in order to asses severity and evolution of their hepatic disease. Informed written consent was obtained from 8 patients (see Table 1). Sections of 4 µm thick were performed by original formalin-fixed, paraffin-embedded tissue blocks. Sections were immunostained for the following antibodies: αSMA (clone αsm-1, Menarini, ready to use), CXCR2 (clone E-2, Santa Cruz, dilution 1∶250) and syndecan-2 (clone 305507, R&D systems, dilution 1∶50). The primary antibody was detected using a biotin-free polymeric-horseradish peroxidase (HRP)-linker antibody conjugate system (Bond Polymer Refine Detection, Leica) conducted with the Bond III automated immunostainer (Leica). The cases illustrated in Figure 6 came from patients 3 and 6 respectively (see Table 1).

### Statistical analysis

All results are expressed as mean ± standard error (SE). Comparisons of more than two groups were made with a one-way ANOVA with post-hoc Tukey's test. Differences were considered statistically significant when P was <0.05.

## Supporting Information

Figure S1 **Image J quantification for immunoblot analyses of** **Figure 1A** **. Data are mean ± SE of 3 independent experiments.**
(TIF)

Figure S2 **Image J quantification for immunoblot analyses of** **Figure 4C** **,** **5A and 5B** **. Data are mean ± SE of 3 independent experiments.**
(TIF)

FigureS3 **Image J quantification for immunoblot analyses of** **Figure 6A and 6B** **. Data are mean ± SE of 3 independent experiments.**
(TIF)
